# Prevalence and treatment coverage for depression: a population-based survey in Vidarbha, India

**DOI:** 10.1007/s00127-016-1220-9

**Published:** 2016-04-22

**Authors:** Rahul Shidhaye, Siddharth Gangale, Vikram Patel

**Affiliations:** Centre for Control of Chronic Conditions, Public Health Foundation of India, 19, Rishi Nagar, Char Imli, Bhopal, Madhya Pradesh India; CAPHRI School for Public Health and Primary Care, Maastricht University, Masstricht, The Netherlands; Institute of Psychiatry, Psychology and Neuroscience, King’s College London, London, UK; International Mental Health and Wellcome Trust Principal Research Fellowship in Tropical Medicine, London School of Hygiene and Tropical Medicine, London, UK; Sangath, Goa, India

**Keywords:** Depression, Treatment coverage, Health services research, India

## Abstract

**Purpose:**

VISHRAM is a community-based mental health program to address psycho-social distress and risk factors for suicide in a predominantly rural population in Central India, through targeted interventions for the prevention and management of Depression and Alcohol Use Disorders (AUD). The evaluation was designed to assess the impact of program on the contact coverage of evidence-based treatments for depression and AUD through a repeated survey design. This paper describes the baseline prevalence of depression among adults in rural community, association of various demographic and socio-economic factors with depression and estimates contact coverage and costs of care for depression.

**Methods:**

Population-based cross-sectional survey of adults in 30 villages of Amravati district in Vidarbha region of Central India. The outcome of interest was a probable diagnosis of depression which was measured using the Patient Health Questionnaire (PHQ-9). Data were analyzed using simple and multiple logistic regression.

**Results:**

The outcome of current depression (PHQ-9 ≥ 10) was observed in 14.6 % of the sample (95 % CI 12.8–16.4 %). The contact coverage for current depression was only 4.3 % (95 % CI 1.5–7.1 %). Prevalence of depression varied greatly between the two sites of the study; higher age, female gender, lower education, economic status below poverty line and indebtedness were associated with depression; and while a contact coverage with formal health care was very low, a large proportion of affected persons had consulted family members.

**Conclusions:**

Our findings clearly indicate that psycho-social distress in rural communities in Maharashtra is strongly associated with social determinants such as gender, poverty and indebtedness and affects the entire population and not just farmers.

**Electronic supplementary material:**

The online version of this article (doi:10.1007/s00127-016-1220-9) contains supplementary material, which is available to authorized users.

## Background

Current efforts in global mental health aim to address the inequities in accessing mental health care within countries and between low-income and high-income countries [1]. The main strategies to achieve this focus on developing, implementing, and evaluating evidence-based practices that can be scaled up through routine health-care platforms [2]. India launched its National Mental Health Program in 1982 with the objective of promoting community participation and accessible mental health services [3]. In practice, though, community mental health programs are very poorly developed and mental health care is not available in primary health care for the vast majority of the population [4]. Maharashtra is the third largest state in India, located in the west of the country. It is a relatively highly industrialized state with a significant urban population (42.5 % of total population) [5]. The state is amongst the leading states in terms of absolute number of suicides [6]. Vidarbha region in the eastern half of Maharashtra state has been in news in last two decades due to the large number of suicides in agricultural communities [7]. It is proposed that these suicides have happened, by and large, due to issues related to agricultural yield, fluctuations in minimum support price, credit availability, income and weather uncertainties [8]. Beyond the problem of suicides in farmers, the findings of the Million Death Study (2012) suggest that suicide mostly kills individuals in their youth, with 40 per cent of suicide deaths in men and 56 per cent of suicide deaths in women occurred at ages 15–29 years, making suicide a leading cause of death in this age group [9]. Mental health conditions are an important proximal risk factor for suicide. By far, the most common mental health conditions which contribute to this risk are depression and alcohol use disorders [10]. It is in this context that the VISHRAM (Vidarbha Stress and Health progRAM) project was launched in November 2011 with the goal of establishing a sustainable rural mental health program to address mental health problems in rural communities in the Vidarbha region of Maharashtra.

The key objective of VISHRAM was to implement and evaluate a comprehensive, population-based, community mental health care program to reduce the psycho-social distress and suicide risk, through targeted interventions for the prevention and management of Depression and Alcohol Use Disorders (AUD). VISHRAM was launched in November 2011 and the mental health care interventions were developed during the period November 2011 to December 2013 (Development Phase). VISHRAM Implementation phase commenced in January 2014 and was completed in October 2015. The evaluation was designed to assess the impact of program on the contact coverage of evidence-based treatments for depression and AUD through a repeated survey design. Contact coverage captures the proportion of persons in need of a service (e.g. the number of cases with a diagnosable disorder such as depression or AUD) who receive an intervention that is appropriate to their condition [11]. The baseline community survey was carried out during the period December 2013–March 2014 and follow-up community survey was completed in August 2015–September 2015.

In this paper, we describe the findings of the baseline community survey for depression. The key research questions addressed in this paper are:

1. What is the prevalence of depression among adults in these rural communities?
2. What is the association of various demographic and socio-economic factors, especially those related to poverty, indebtedness, and distress selling/mortgaging of land with depression in adults?
3. Where do people seek care for depression and what is the estimate of contact coverage?
4. What is the cost of care for depression in this population?

## Methods

### Study design

Population-based cross-sectional survey of adults.

### Setting

This cross-sectional survey was conducted in all the 30 villages where VISHRAM was implemented during 2013–2015 (Implementation Phase). Prakriti, a Non-Governmental Organization (NGO) working in the social development sector, and one of the partners in VISHRAM had recent or ongoing livelihood programs in these villages. Half of these villages (*n* = 15) were in Chandur Bazaar (pop 49,451) and half in Dhamangaon taluka (pop 51,104) of Amravati District in Vidarbha region. A taluka is an administrative block in a district. Amravati district has 14 talukas with district headquarters located in Amravati city. In the city of Amravati there is one medical college and one district hospital in public sector and they provide tertiary care. The District hospital has inpatient facilities with 373 beds and the District Mental Health Program (DMHP) is based in this hospital. There is one psychiatrist in the District Hospital who leads DMHP in addition to one psychologist, one occupational therapist and two psychiatric social workers. There are ten rural hospitals in the district with a total capacity of 270 beds, 56 Primary Health Centers (PHCs) and 333 sub-centers. There are no psychiatrists or psychologists in public as well as in private sector in Chandur Bazaar and Dhamangaon town as well as in these talukas. Mental health services are available only in the District Hospital in the public sector and neither rural hospitals nor PHCs provide any of these services. There are around 15 psychiatrists in private sector and all are based in Amravati city. Traditional healers also provide mental health services and they are located across the district.

### Sample selection

The survey was conducted during a 4 month period from December 2013 to March 2014. Systematic random sampling was used to select the participants from voter lists in all the 30 villages; thus the sampling frame comprised all adults aged >18 years. The voter lists were obtained from the website of the Election Commission which had been updated in 2013 [12]. Our sample size calculation was based on the estimated effect of the intervention on contact coverage in adults with depression or AUD. Our target was 1516 individual and, to account to an estimated 20 % non-participation rate, we randomly selected 1900 individuals from the voter list. Recruitment of participants was conducted by trained field researchers through face-to-face interviews.

Eligibility criteria for participation was requirement to be fluent in Marathi and absence of any cognitive impairment which was severe enough to interfere with the informed consent procedure or survey (for, e.g. severe intellectual disability). All adults received a verbal introduction to the study by the field researcher and were provided with an information sheet, and then approached for consent to participate in the study.

### Measures

A structured interview schedule was developed specifically for the survey and modelled on a schedule used in another cross-sectional community survey conducted as part of the PRIME project in the neighboring state of Madhya Pradesh [13]. The interview schedule was first designed in English and then translated in Marathi and piloted before use in the survey. The interview schedule contained sections on: socio-demographic characteristics (details below); inpatient and outpatient health care service utilisation; screening for depression using PHQ-9; help-seeking for depression (if screened positive); screening for AUD using AUDIT; help-seeking for AUD (if screened positive); suicidality; mental health-related knowledge, attitudes and behaviours; and disability. This structured interview was administered by field researchers whose minimum qualification was a college degree. They received a week long intensive training led by an experienced researcher. The field researchers were recruited only for baseline survey and were independent of VISHRAM implementation team. An overview of VISHRAM project was included in their training but they were not aware of the detailed intervention packages.

The following explanatory variables factors were explored in the analyses presented in this paper, based on our hypotheses:

*Demographic factors *The taluka, age, gender, education, marital status, religion and caste. Education was treated as a categorical variable with five levels; the caste variable had four levelsscheduled caste, scheduled tribe, other backward caste and general (neither scheduled caste/scheduled tribe nor other backward caste). Scheduled castes and scheduled tribes are groups recognized as socially and economically disadvantaged.

*Socio-economic factors* Occupation, annual household income, type of house, ownership of land, indebtedness, if they had to sell/mortgage land due to economic reasons, possession of Below Poverty Line (BPL) card and employment with Mahatma Gandhi National Rural Employment Guarantee Act (MNREGA). Type of house was categorized in a similar way as done in National Family Health Survey-III [14]. ‘Kachha’ houses were made from mud, thatch, or other low-quality materials, ‘Semi-pucca’ houses used partly low-quality and partly high-quality materials and houses made with high quality materials throughout, including the floor, roof, and exterior walls, were categorized as ‘pucca’ houses. Below Poverty Line is an economic benchmark and poverty threshold used by the Government of India to identify individuals and households in need of government financial assistance. It is generally based on minimum expenditure per capita necessary to survive [15]. BPL card is issued to all the households below the income threshold. MNREGA is a national scheme which aims at enhancing the livelihood security of people in rural areas by guaranteeing 100 days of wage-employment in a financial year to a rural household whose adult members volunteer to do unskilled manual work [16].

The outcome of interest was a probable diagnosis of depression which was measured using the Patient Health Questionnaire (PHQ-9). PHQ-9 is a screening questionnaires widely used in research and practice to screen patients for depression [17]. A cut-off score of 10 or higher is found to have a sensitivity of 88 % and a specificity of 88 % for detecting depression [18]. A recent systematic review further supports the psychometric properties of PHQ-9 and reports that there are no significant differences in sensitivity or specificity at a cut-off score of 10 compared with other cut-off scores within the interval (8–11) [19]. In our study we used the Marathi version of PHQ-9 which has been validated elsewhere in India [20, 21]. Participants who screened positive on PHQ-9, i.e. who scored >9, were further asked questions related to seeking help for the problems due to depression from a range of providers, including non-formal providers. Details about the treatment received, medications and costs of care were also addressed. In the end, participants were also asked questions on suicidal ideation which was adapted from the Mini International Neuropsychiatric Interview (MINI), a semi-structured interview for the assessment of mental disorders.

### Analysis

The association of each demographic and socio-economic risk factor with the outcome of depression was first assessed using logistic regression (univariable analysis). All factors whose association reached significance at *p* ≤ 0.05 in the univariable analysis were included in multivariable analysis. Gender and taluka (Chandur Bazaar/Dhamangaon) were included as a priori variables in the multivariable model. Retention of other variables in the model was based on forward selection and likelihood ratio tests. We report odds ratios (ORs) and 95 % confidence intervals (CIs) for all associations. Data were analysed using STATA/IC version 11.2.

## Results

### Characteristics of the sample

Of the 1900 participants randomly selected, 444 (23.4 %) could not participate for the following reasons: inability to locate the individuals who either migrated permanently or temporarily, death, refusal to participate in survey and inability to consent. The final sample comprised of 1456 individuals between the ages of 18–87 years old with an average age of 43.3 years (SD ± 15.5). Just over half were male (52.3 %). 14.1 % of the sample was illiterate. These figures are very similar to the overall population characteristics of Amravati district [22]. Table 1 provides comparison of socio-demographic factors between Dhamangaon and Chandur Bazaar taluka.

**Table 1:** Comparison of Socio-demographic factors between Dhamangaon and Chandur Bazaar taluka

Socio-demographic factor	Prevalence of the socio-demographic factor *n* (%)		*p* value
	Dhamangaon	Chandur bazaar	
Age (in years)			0.771
18–30	186 (28.0)	214 (27.0)	
31–40	168 (25.3)	187 (23.6)	
41–55	158 (23.8)	202 (25.5)	
>55	152 (22.9)	189 (23.9)	
Gender			0.268
Male	337 (50.8)	425 (53.7)	
Female	327 (49.2)	367 (46.3)	
Education			0.015
Graduation and above	43 (6.5)	73 (9.2)	
Junior college	113 (17.0)	95 (12.0)	
High school (8–10)	196 (29.5)	232 (29.3)	
Primary and middle school	212 (31.9)	286 (36.1)	
Illiterate	100 (15.1)	106 (13.4)	
Occupation (*n* = 1415)			0.011
Professional	48 (7.3)	35 (4.6)	
Manual laborer	17 (2.6)	30 (3.9)	
Agriculture	427 (65.0)	455 (59.9)	
Household work	124 (18.9)	178 (23.4)	
Unemployed	40 (6.1)	61 (8.0)	
Annual income (*n* = 1434)			<0.001
First quintile	79 (12.0)	212 (27.2)	
Second quintile	121 (18.4)	212 (27.2)	
Third quintile	90 (13.7)	189 (24.3)	
Fourth quintile	198 (30.1)	109 (14.0)	
Fifth quintile	169 (25.7)	57 (7.3)	
Employed with MNREGA (*n* = 1360)			0.347
Yes	58 (9.5)	83 (11.0)	
No	553 (90.5)	668 (88.9)	
Below poverty line card (*n* = 1449)			0.469
No	358 (54.2)	414 (52.3)	
Yes	302 (45.8)	377 (47.7)	
Marital status			<0.001
Unmarried	60 (9.0)	112 (14.1)	
Currently married	570 (85.8)	611 (77.1)	
Religion (*n* = 1453)			<0.001
Hindu	547 (82.5)	550 (69.4)	
Muslim	51 (7.7)	125 (15.8)	
Neo-Buddhist and others	65 (9.8)	117 (14.8)	
Caste (*n* = 1445)			<0.001
OBC	373 (56.9)	419(52.9)	
Schedule caste	101 (15.4)	183 (23.1)	
Schedule tribe	74 (11.3)	36 (4.5)	
General	107(16.3)	154 (19.4)	
House type (*n* = 1436)			0.005
Kuchha	236 (36.5)	335 (42.3)	
Semi-pucca	261 (40.4)	255 (32.2)	
Pucca	149 (23.1)	202 (25.5)	
Land ownership			<0.001
Yes	615 (92.6)	670 (84.6)	
No	49 (7.4)	122 (15.4)	
Loan (*n* = 1453)			0.762
No	562 (84.6)	674 (85.2)	
Yes	102 (15.4)	117 (14.8)	
Loan amount in Rupees (*n* = 217)			0.001
9000–15000	27 (26.7)	26 (22.4)	
17000–25000	19 (18.8)	20 (17.2)	
26000–40000	26 (25.7)	23 (19.8)	
45000–65000	22 (21.8)	13 (11.2)	
70000 and above	7 (6.9)	34 (29.3)	
Sold or Mortgaged land (*n* = 1446)			0.001
No	600 (90.9)	750 (95.1)	
Yes	60 (9.1)	38 (4.8)	
Settled loan (*n* = 212)			0.790
Yes	23 (22.8)	27(24.3)	
No	78 (77.2)	84(75.7)	

*n* = 1456 unless specified otherwise

The outcome of current depression was observed in 14.6 % of the sample (95 % CI 12.8–16.4 %; *n* = 212). There was marked variation in prevalence of current depression in two talukas; the prevalence was much higher in Chandur Bazaar (19.5 %, 95 % CI 16.7–22.3 %) compared with much lower in Dhamangaon (8.7 %, 95 % CI 6.6–10.9 %). The prevalence of severe depression (defined as PHQ9 ≥ 15) was 3.9 % (95 % CI 2.9–4.8 %) with again a marked inter-site variation (Chandur Bazaar: 6.2 %,95 % CI 4.5–7.9 %; Dhamangaon: 1.1 %, 95 % CI 0.3–1.8 %).

### Demographic and socio-economic correlates of depression

In the final multivariable analysis, age, gender, education, poverty (households with below poverty line card) and indebtedness along with place of residence (taluka) retained significant association with the outcome of current depression (Table 2). Elderly, especially above 55 years of age had seven times higher odds of depression compared to young adults. Females were 40 % more likely to have depression compared to males, while an inverse association was observed between education and depression. The risk of depression was one and half times in individuals belonging to households below poverty line and it was double in those who were in debt. Even after adjusting for the demographic and socio-economic factors, the odds for depression were two and half times in individuals from Chandur Bazaar taluka compared with those from Dhamangaon taluka.

**Table 2:** Association of socio-demographic factors with prevalence of current depression (PHQ9 >=10)

Socio-demographic factor	Prevalence of the socio-demographic factor *n* (%)	Prevalence of current depression *n* (%)	Unadjusted odds ratio with 95 % CI	Adjusted odds ratio with 95 % CI
Taluka				
Dhamangaon	664 (45.7)	58 (8.7)	1	1
Chandur bazaar	792 (54.3)	154 (19.5)	2.5 (1.8–3.5)	2.7 (1.9–3.8)
Age (in years)				
18–30	400 (27.5)	22 (5.5)	1	1
31–40	355 (24.4)	29 (8.2)	1.5 (0.9–2.7)	1.5 (0.8–2.7)
41–55	360 (24.7)	52 (14.5)	2.9 (1.7–4.9)	2.6 (1.5–4.5)
>55	341 (23.4)	109 (32)	8.1 (5.0–13.1)	6.8 (3.9–12)
Gender				
Male	762 (52.3)	116 (12.6)	1	1
Female	694 (47.7)	96 (16.7)	1.4 (1.0–1.9)	1.4 (1.0–2.0)
Education				
Graduation and above	116 (8.0)	6 (5.2)	1	1
Junior college	208 (14.3)	11 (5.3)	1.0 (0.4–2.8)	1.1 (0.4–3.2)
High school (8–10)	428 (29.4)	61 (14.3)	3.1 (1.3–7.3)	2.5 (1.0–6.1)
Primary and middle school	498 (34.2)	78 (15.7)	3.4 (1.4–8.0)	1.6 (0.6–4.0)
Illiterate	206 (14.1)	56 (27.3)	6.9 (2.9–16.6)	2.5 (1.0–6.4)
Occupation (*n* = 1415)				
Professional	83 (6)	6 (7.2)	1	
Manual laborer	47 (3.3)	2 (4.3)	0.6 (0.1–2.9)	
Agriculture	882 (62.3)	122 (13.8)	2.1 (0.9–4.8)	
Household work	302 (21.3)	54 (17.8)	2.8 (1.2–6.7)	
Unemployed	101 (7.1)	24 (23.8)	4 (1.5–10.3)	
Annual income (*n* = 1434)				
First quintile	291 (20.3)	57 (20)	1	
Second quintile	333 (23.2)	57 (17.1)	0.8 (0.6–1.3)	
Third quintile	277 (19.4)	45 (16.3)	0.8 (0.5–1.2)	
Fourth quintile	307 (21.4)	27 (8.8)	0.4 (0.2–0.6)	
Fifth quintile	226 (15.7)	23 (10.2)	0.5 (0.3–0.8)	
Employed with MNREGA (*n* = 1360)				
Yes	141 (10.4)	26 (18.4)	1	
No	1219 (89.6)	172 (14.1)	0.7 (0.5–1.1)	
Below poverty line card (*n* = 1449)				
No	771 (53.2)	90 (11.7)	1	1
Yes	678 (46.8)	122 (18.0)	1.7 (1.2–2.2)	1.5 (1.1–2.1)
Marital status				
Unmarried	172 (11.8)	11 (6.4)	1	
Currently married	1181 (81.2)	164 (13.9)	2.4 (1.3–4.4)	
Widow/divorced/separated	103 (7)	37 (36.3)	8.3 (4–17.3)	
Religion (*n* = 1453)				
Hindu	1095 (75.4)	163 (15)	1	
Muslim	176 (12.1)	12 (7)	0.4 (0.2–0.8)	
Neo-Buddhist and others	182 (12.5)	37 (20.3)	1.5 (1.0–2.2)	
Caste (*n* = 1445)				
OBC	791 (54.7)	122 (15.4)	1	
Schedule caste	283 (19.6)	56 (20)	1.4 (0.9–1.9)	
Schedule tribe	110 (7.6)	7 (6.4)	0.4 (0.2–0.8)	
General	261 (18.1)	26 (10)	0.6 (0.4–0.9)	
House type (*n* = 1436)				
Kuchha	570 (39.7)	104 (18.3)	1	
Semi-pucca	515 (35.9)	49 (9.5)	0.4 (0.3–0.7)	
Pucca	351 (24.4)	59 (16.8)	0.9 (0.6–1.3)	
Land ownership				
Yes	1285 (88.4)	190 (14.8)	1	
No	171 (11.6)	22 (13)	0.9 (0.5–1.4)	
Loan (*n* = 1453)				
No	1234 (84.9)	164 (13.3)	1	1
Yes	219 (15.1)	48 (22)	1.8 (1.3–2.6)	1.8 (1.2–2.7)
Loan amount in rupees (*n* = 217)				
9000–15,000	53 (24.4)	7 (13)	1	
17,000–25,000	39 (17.9)	11 (28)	2.5 (0.9–7.4)	
26,000–40,000	49 (22.6)	6 (12)	0.9 (0.3–2.9)	
45,000–65,000	35 (16)	10 (29)	2.6 (0.9–7.8)	
70000 and above	41 (18.9)	14 (34)	3.4 (1.2–9.5)	
Sold or mortgaged land (*n* = 1446)				
No	1348 (93.2)	200 (14.8)	1	
Yes	98 (6.8)	11 (11.2)	0.7 (0.4–1.4)	
Settled loan (*n* = 212)				
Yes	50 (23.6)	7 (14)	1	
No	162 (76.4)	40 (24.7)	2 (0.8–4.8)	

*n* = 1456 unless specified otherwise

Of all the individuals interviewed, 75 (5.2 %) responded that they have thought of taking their life in last 12 months. Close to half of them (45.3 %) also had current depression and there was no significant variation across talukas, age categories or sex. Out of the 75 individuals, 4 (5.3 %) had made a plan for committing suicide in last 12 months. Most of them had current depression (*n* = 3). None of the individuals interviewed reported a suicide attempt in last 12 months.

### Help-seeking and treatment coverage

Half of the individuals with current depression had spoken about their problems with at least one person in their social network. Of those who had spoken about their problem, 71 % discussed the problem with their spouse and 48.1 % discussed this with their children. However, a very small proportion of individuals (*n* = 9, 4.3 %) had sought formal health care for problems related to current depression. There was again a marked difference between the two talukas; 13.8 % (95 % CI 4.6–22.9 %) in Dhamangaon compared with just 0.7 % (95 % CI 0.001–2.1 %) in Chandur Bazaar.

The general physician was consulted by eight and only one individual consulted a specialist. Medications were prescribed to six and three received some form of counseling. A third of these individuals (*n* = 3) reported lot of help due to these treatments, while the remainder reported little or no help. Treatment was being continued by six individuals; one had stopped as there was no improvement while two stopped as treatment was costly.

The estimates for treatment costs for last 12 months are based on the data from these nine individuals and hence show a wide variation. The total cost of treatment varied from USD 81.8 to 4147.2 with a mean of USD 1650.3 (standard deviation: 1296.8) and median of USD 1244.2. Consultation fees constituted half of the median costs (USD 701) while medications (median: USD 137.2) and for travel (median: USD 175.2) approximately accounted for a quarter of the expenses. The median per capita income in rural India is USD 275.2 while it is slightly higher in rural Maharashtra (USD 311.7) [23].

## Discussion

We report the findings of a population based survey of the prevalence, socio-economic risk factors and help-seeking for depression in a rural population in India. Our main findings are that the prevalence of depression varied greatly between the two sites of the study; higher age, female gender, lower education, economic status below poverty line and indebtedness were associated with depression; and while a contact coverage with formal health care was very low, a large proportion of affected persons had consulted family members. Almost all who went to a health care provider had consulted a general physician. The median costs of care for the episode was equivalent to four and half times the median rural per capita income of the country [23].

There is a wide variation in prevalence of depression in India with figures in the range of 2–57 % [24] and one meta-analysis reports a pooled prevalence of 8.9 % for depression [25], a figure that closely approximates our overall prevalence estimate, although it varied over two fold across the two talukas. This variation was observed despite the same research team and methodology and timing of the survey. The findings from the World Mental Health Surveys has also shown that the prevalence of depression and other common mental disorders varies widely between populations cross-nationally [26, 27]. Our prevalence estimates are also higher than those reported by Ferrari et al. from the Global Burden of Disease 2010 study [28] as well as the rates reported by Bromet et al. from the World Mental Health Survey [27]. Estimates from these studies are based on assessment of depression using structured diagnostic tool such as Composite International Diagnostic Interview (CIDI), while our estimates are based on use of screening tool (PHQ-9) which might possibly explain some of these differences. However, a large community-based study from South India using a modified PHQ-9 has observed 15.1 % prevalence of depression which is very close to our overall estimate [29] and, further, the higher prevalence observed in our study may also be a true reflection of the higher prevalence of risk factors associated with the condition. The strong independent association of site with depression after adjusting for demographic and socio-economic factors suggests that other unknown ecological factors are key drivers for depression.

We observed a monotonic increase in prevalence of depression with age which is contrary to the literature from high-income countries [30] which suggest that depression peaks in middle age. However, our findings are consistent with other studies from India [29, 31]. This difference in the age distribution requires further study and may indicate a higher prevalence of risk factors in older people such as chronic diseases. It is proposed that structured, complex interview schedules such as Composite International Diagnostic Interview (CIDI) tend to underestimate prevalence of mental disorders in older age groups compared to screening tools (GHQ-12, K10) [32], and this might possibly explain the increased prevalence of depression in older age groups we observed in our study. Our findings of a higher risk in women, however, is consistent with the global and contextual literature [29, 33, 34]. Our findings support the robust evidence regarding the association of lower socio-economic position with depression. There is high level of inequity in the distribution of CMDs across socio-economic strata within societies, with significantly increased rates of depression among lower socio-economic groups [35–37]. Our finding of independent associations between lower levels of education, living below poverty line and indebtedness supports this observation. However, we did not observe any association between being a member of a disadvantaged sub-group (Scheduled castes and tribes) and depression. Importantly, in the context of the ongoing concerns about the mental health of farmers we did not observe any association between agricultural occupations and depression. Nevertheless, suicides in farmers needs to be explored further. Two studies based on psychological autopsies of farmer’s suicides found that indebtedness and hopelessness due to crop failure leads to decline in economic status resulting in family disputes, depression and drinking problems [7, 38]. This, coupled with easy access to pesticides and government compensation following death due to suicide, creates a toxic brew of circumstances which ultimately lead to farmers attempting suicide.

The contact coverage for depression was only 5 % but over half of affected persons had discussed their problems with someone in their social network (e.g. spouse, children, friends etc.). Furthermore, most of the individuals who did seek care visited a general physician. Only one individual had sought care from psychiatrist and none had approached community health workers or para-medical staff in public health sector. Our findings are consistent with those observed in a multi-centric epidemiological study of mental disorders conducted by the Ministry of Health which found that only 5.1 % people with mental disorders in past 12 months had utilized mental health services and were provided a prescriptive treatment. A study conducted as part of the World Health Survey has reported 12.5 % coverage for people with depression [39]. The large difference in the contact coverage observed between two sites could be potentially explained by the fact that villages in Dhamangaon taluka have good transportation and access to specialist (psychiatrist) services available in two medical colleges providing tertiary care in Wardha city which is the neighboring district headquarter. One of these medical colleges, Datta Meghe Institute of Medical Sciences provides free transportation to patients (including those with mental disorders) in adjoining rural areas including villages in which VISHRAM was implemented. Thus, for patients with depression in Dhamangaon taluka, services are available in two cities (Amravati and Wardha) compared to only one (Amravati) for those in Chandur Bazaar taluka. In addition, transportation related barriers associated with ‘access to care’ are also addressed resulting in better contact coverage.

Conceptualization of mental illness and beliefs in effectiveness of treatment modalities influence help-seeking for depression [40]. In India, and particularly in rural communities, mental disorders are conceptualized as equivalent to psychosis and epilepsy and the symptoms associated with depression are perceived as secondary to the social and economic problems [41]. Non-recognition of these symptoms as a medical condition (depression) as well as stigma attached to labels related to mental disorders are likely to be important reasons for the low levels of contact coverage. Availability of services also impacts help-seeking behavior as described above. Currently the specialist services are available only at the district headquarters [Amravati and Wardha (for Dhamangaon taluka)] which plays a significant role in delaying help-seeking and general physicians in public as well as private sector do not explicitly provide services for common mental disorders. In this context, community-based health workers could play an important role in as they are ideally placed to identify people experiencing depression, provide low intensity psychosocial interventions, make appropriate referrals to primary care, and contribute to raising awareness about depression being a treatable condition.

Other studies from India have demonstrated that individuals with depression spend more days being unable to work as usual due to their illness [42]. A study in India in primary care estimated the cost of an episode of a common mental disorder to be equivalent to 3 weeks’ wages for agricultural workers [43]. A population based study of the health care costs of three common conditions affecting women (reproductive tract infections, anaemia and depression) reported that only depression was associated with increased health care costs and markedly increased the risk of catastrophic health expenditure [44]. The data from our study on costs of care for depression in rural settings in very limited as only 9 individuals had accessed services for their complaints related to depression. The median cost of care was very high and was primarily for the consultation fees of the doctor indicating the use of private practitioners for depression. Very high costs of care is are potentially an important barrier to access health services resulting in low contact coverage. Lack of adequate protection against financial risks leading to catastrophic health expenditures pushes millions of Indians into poverty trap [45] which essentially underlines the importance of integrating mental health care in the public health care system where the costs incurred by patients are primarily related to travel and medications.

As with all cross-sectional surveys, our study is not able to unpack the causal inferences about the associations we observed. Importantly, while we observed a large variation in prevalence across the two talukas, the assessments we carried out were unable to explain this. Variables specifically related to women’s mental health such as husband’s alcohol intake, and inter-personal violence were not part of the questionnaire. Physical health is also associated with depression, but was also not assessed. We are unable to comment on effective coverage and we also have limited data on cost of care for depression from this survey.

Notwithstanding these limitations, this paper describes one of the few studies from India which attempts to assess the treatment coverage of services for depression in rural settings. There are also very few recent studies from rural India which have assessed association of various socio-economic factors with depression. Thus, this study does address key research questions on the epidemiology of depression in rural India.

Our findings clearly indicate that psycho-social distress in rural communities in Maharashtra is strongly associated with social determinants such as gender, poverty and indebtedness and affects the entire population and not just farmers. Although depression is a common condition, there are wide small area variations which require explanation. Treatment gaps are very high and it is essential to implement community based mental health programs which address both the demand side and supply side barriers which contribute to low levels of contact coverage. This is the goal of the VISHRAM program and the findings of the repeat survey in the same population will be reported in due course.

## Electronic supplementary material

Below is the link to the electronic supplementary material.

Supplementary material 1 (XLSX 17 kb)
